# Long-Term Evolution of the Hip and Proximal Femur after Hip Reconstruction in Non-Ambulatory Children with Cerebral Palsy: A Retrospective Radiographic Review

**DOI:** 10.3390/children9020164

**Published:** 2022-01-28

**Authors:** Norine Ma, Peter Tischhauser, Carlo Camathias, Reinald Brunner, Erich Rutz

**Affiliations:** 1 Department of Orthopaedics, The Royal Children’s Hospital, Melbourne 3052, Australia; norinemaau@gmail.com; 2 Department of Pediatric Orthopaedics, University Children’s Hospital Basel (UKBB), 4056 Basel, Switzerland; tischhauser.p@gmail.com (P.T.); reinaldbrunner@sunrise.ch (R.B.); 3 Praxis Zeppelin, 9016 St. Gallen, Switzerland; Camathias.carlo@gmail.com; 4 Medical Faculty, University of Basel, 4001 Basel, Switzerland; 5 Department of Paediatrics, The University of Melbourne, Melbourne 3010, Australia; 6 Murdoch Children’s Research Institute, Melbourne 3052, Australia

**Keywords:** hip reconstruction, hip dislocation, cerebral palsy, non-ambulatory, femoral head deformity

## Abstract

Background: Hip displacement in children with cerebral palsy (CP) has a higher prevalence in non-ambulatory children. Progression can lead to pain, pelvic obliquity and difficulty with sitting. This can be addressed with hip reconstruction. Our study aims to report the long-term radiological outcomes after hip reconstruction, in particular the evolution of femoral head deformity. Methods: A total of 58 hips of non-ambulatory children with CP were evaluated retrospectively using pre-operative, early (median 120 days) and late post-operative (median 8.6 years) anteroposterior standardised radiographs. All the hips were treated with femoral shortening varus derotation osteotomy (VDRO), pelvic osteotomy and an open reduction, if indicated. The radiographical indices measured included the migration percentage (MP), sharp angle, acetabular index, centre-edge angle, neck shaft angle, head shaft angle, pelvic obliquity, femoral head sphericity, femoral head deformity (FHD) and growth plate orientation. Results: Improvements in hip congruency and morphology were evident after reconstructive hip surgery. These were maintained at the late post-operative time point. Median MP improved from 56% (IQR 46–85%) to 0% (IQR 0–15%) at early follow-up. This increased to 12% (IQR 0–20%) at late follow-up. Pre-operatively, FHDs of 14 hips (24%) were classified as grade A (spherical femoral head). This increased to 22 hips (38%) at early follow-up and increased further to 44 hips (76%) at late follow-up. Conclusions: Our study shows that hip reconstruction reduces hip displacement in the long term, indicated by decreased post-operative MP maintained at long-term follow-up. Although non-ambulatory children lack weight-bearing forces promoting bone remodelling, improved femoral head morphology after surgery alters the forces between the acetabulum and the femoral head. Mild femoral head deformity (grades A and B) remained stable and even improved after surgery, postulated to be due to severe osteoporosis allowing remodelling.

## 1. Introduction

Cerebral palsy (CP) results from non-progressive brain injury that can cause progressive musculoskeletal pathology, such as muscle contractures and bone deformities [1]. Brain dysfunction and deformities can result in functional impairment, which is described using the Gross Motor Function Classification System (GMFCS) [2]. Hip displacement commonly occurs in children with CP and is thought to be related to abnormal forces at the hip joint exerted by the surrounding muscles [3]. It has a higher prevalence in non-ambulatory children (GMFCS levels IV and V) and those with a greater neurological involvement, such as spastic quadriplegia [4]. While hip displacement is often asymptomatic in the early stages, progression can lead to pain, scoliosis and functional impairment, such as difficulties with sitting in GMFCS IV and V [5].

Various hip surveillance programs have been suggested for the early detection of hip displacement using radiographs at specified intervals according to risk [6,7]. These recognise the increased risk of progression in non-ambulatory children, with shorter screening intervals recommended for those with higher GMFCS levels [6,7]. Early detection allows for interventions prior to the development of symptoms and the need for salvage procedures. Before surgical intervention is required, bracing, physiotherapy and botulinum toxin A injections to the hip adductors can be considered to prevent or slow down the progression of hip displacement [8,9]. Surgical interventions include preventative soft tissue and bony procedures, including hip reconstruction, which have been shown to result in satisfactory pain relief and good radiological outcomes [10]. Salvage procedures may be indicated when there is a severe degeneration of the hip joint, but carries a higher risk for ongoing pain and heterotopic ossification [11].

Children with CP can also have deformities of the proximal femur, including femoral neck anteversion and increased neck shaft angle [12]. An increased neck shaft angle has been associated with instability of the hip [13]. In non-ambulatory children, it has been postulated that the deficiency of weight-bearing during early development is associated with the lack of proximal femur remodelling as is found in ambulatory children. However, Rutz et al. [14] reported that hip reconstruction is effective in improving hip congruity even when the femoral head is deformed, and it has been proposed that the femoral head is able to remodel itself after hip reconstruction [1,15].

Many radiographic indices have been used in measuring hip displacement and associated features, such as acetabular dysplasia. Although the use of two-dimensional radiographs in visualising the hip joint comes with its limitations, radiographic features are used as surrogate measures for determining the success of hip reconstruction surgeries [14,15,16]. The severity of hip displacement is commonly measured using Reimers’ migration percentage, as it has been shown to have good intra- and inter-observer reliability and is not greatly impacted by the rotation of the femur [17,18].

This study focusses on the radiological outcomes of non-ambulatory children after hip reconstruction, in particular any remodelling of the proximal femur.

## 2. Materials and Methods

### 2.1. Study Design

The radiographs of non-ambulatory patients (GMFCS levels IV and V) with CP before and after hip reconstruction surgery were evaluated retrospectively, with approval from the local ethics committee (EKNZ 2015/224). All the radiographs were anteroposterior supine hip Xa-rays taken using a standardised method. Consecutive patients were selected from one institution (University Children’s Hospital Basel—UKBB) from 1989 to 2005.

Inclusion criteria: Patients were included if they had a confirmed diagnosis of CP; had undergone hip reconstruction surgery due to hip dislocation or subluxation (MP > 40% or MP < 40% with painful anterior or posterior hip dislocation confirmed by CT); radiographs were available pre-operatively, at early post-operative follow-up, and at least five years post-operatively; and were non-ambulatory, defined by Gross Motor Function Classification System (GMFCS) levels IV and V. Exclusion criteria: Patients were excluded if a complete dataset was unable to be obtained due to missing radiographs or the inadequate imaging of the complete acetabulum and proximal femur.

The patient demographic data obtained included sex, side of hip on which the surgery was performed, unilateral or bilateral CP, GMFCS level, age at operation and dates of each radiograph.

### 2.2. Surgery

Pre-operative radiographs in anteroposterior and Dunn–Ripstein views were utilised in conjunction with three-dimensional computed tomography (CT) for surgical planning and determining the choice of internal fixation plate. General anaesthetic and antibiotic prophylaxis were used for all surgeries.

Hip reconstruction surgery consisting of femoral derotation, shortening and varus osteotomy (VDRO), followed by pelvic osteotomy (modified Pemberton), and open reduction, if required, were performed according to the technique described by Brunner and Baumann [19]. The femur was approached using a lateral incision. Kirschner wires were used to determine the degree of femoral anteversion with the aid of an image intensifier control. Oblique femoral osteotomy with pre-determined dimensions was performed in one piece with a defined amount of shortening. A Synthes blade was inserted with an aim to achieve a femoral neck-shaft angle of 120 to 125 degrees, and femoral anteversion of 20 to 25 degrees. This was secured with Kirschner wire if necessary.

Intra-operative arthrography was conducted to determine the need for open reduction using the Smith–Peterson approach [20]. Pelvic osteotomy was performed according to the level determined by the insertion of a Kirschner wire with an image intensifier control. This was performed in a posterior direction. A Hohmann retractor was used to protect the sciatic nerve. The bone block from the femur was cut into wedges and inserted to stabilise the reduced hip joint. Soft tissue procedures (e.g., the lengthening of the hip adductors) were added if indicated.

Post-operatively, a spica cast was applied for 6 weeks for pain relief and to avoid the development of contractures. Sitting was allowed once a hip flexion of 90 degrees was achieved. Standardised radiographs were obtained at follow-up visits and hardware was removed at least six months after the initial surgery.

### 2.3. Measurements

Radiological indices were measured on pre-operative, early post-operative and late post-operative radiographs using a standardised method.

#### 2.3.1. Hip Displacement

The displacement of the femoral head was measured using Reimers migration percentage (MP) [21]. The apex of the gothic arch was used as the lateral point of the acetabulum when present. The MP formed a part of the determination of the revised Melbourne Cerebral Palsy Hip Classification score [22]. The coverage of the femoral head by the acetabulum was also measured using the modified CE angle [23], as the original CE angle is inaccurate in cases of hip displacement due to its use of a line between the femoral heads as a reference point. The modified CE angle uses a horizontal line that takes into account the obliquity of the pelvis. The value of the CE angle considered to be normal depends on the age of the patient, with modified CE angles above 20 degrees considered to be normal from age 3 and above [21].

#### 2.3.2. Pelvis and Acetabulum

Two methods were used to measure the development of the acetabulum. The acetabular index (AI) was measured relative to Hilgenreiner’s line [24]. However, this is known to be affected by kyphosis and lordosis. The sharp angle was measured relative to the pelvic teardrops [21].

The pelvic obliquity (PO) was measured using the inferior tips of the pelvic tear drops as a reference, which has been previously shown to have excellent reliability and validity [25]. The clockwise rotation was measured as positive values, while the anticlockwise rotation was designated negative values.

#### 2.3.3. Sphericity and Femoral Head Deformity

**Figure 1:**
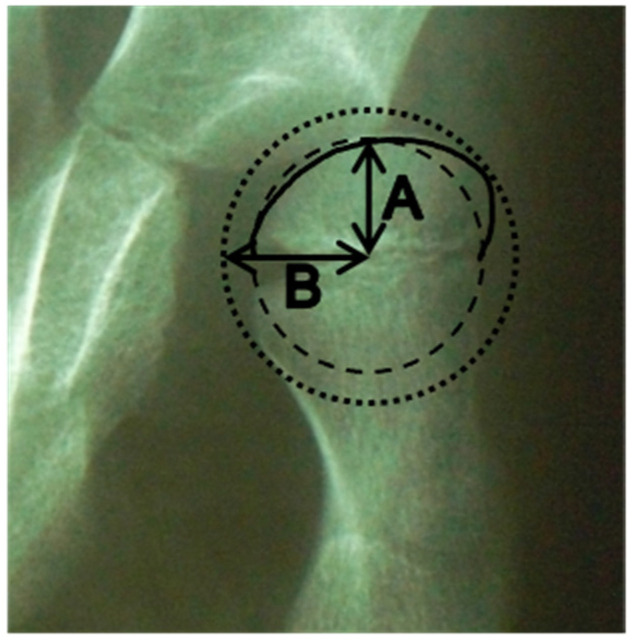
Measurement technique of the femoral head sphericity. A indicates the radius of the smallest circle that fits within the contour of the femoral head. B indicates the radius of the largest circle which encloses the femoral head. The ratio is calculated by A divided by B.

The femoral head deformity (FHD) was classified from grade A to D, according to visual inspection of the femoral head shape on the anteroposterior radiograph [14]. A collapsed femoral head could not be classified using the femoral head deformity grade classification and was considered to be avascular necrosis (AVN). The femoral head sphericity was measured using a previously described modified Mose technique [15], with ratios closer to 1 indicating a more spherical head [Figure 1].

The modified neck shaft angle (mNSA) was measured using the apex of the lesser trochanter of the femur as the point of reference. This has been shown to be reproducible [26]. The head shaft angle (HSA) was measured using a line perpendicular to the growth plate, against the line bisecting the femoral neck [27]. The mNSA and HSA were only measured in pre-operative and late post-operative radiographs, as the early post-operative radiographs were found to have an insufficient coverage of the distal femur for measuring the shaft axis.

**Figure 2:**
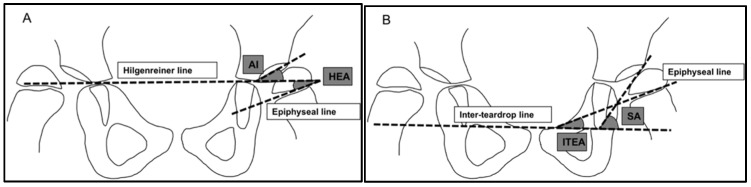
Angles used for measuring the orientation of the femoral growth plate. (**A**) Angles used to measure the acetabular-epiphyseal angle. (**B**) Angles used to measure the difference between the inter-teardrop epiphyseal angle and Sharp angle. AI = acetabular index. HEA = Hilgenreiner epiphyseal angle. ITEA = inter-teardrop epiphyseal angle. SA = Sharp angle.

Two methods of measuring the orientation of the growth plate in relation to the acetabulum were used. The acetabular-epiphyseal angle (AEA) was measured by subtracting the Hilgenreiner epiphyseal angle (HEA) from the AI. This has previously been shown to be correlated with MP in a preliminary study [28]. We also measured the difference between the angle between the growth plate and the inter-teardrop line, and the Sharp angle [Figure 2].

#### 2.3.4. Statistics

The normality of each variable was tested using the visual inspection of the shape of the distribution and the Shapiro–Wilk test. When the variables had a normal distribution, paired *t*-tests were used to test for the differences between the pre-operative, early post-operative and late post-operative values for each variable. In variables that were not normally distributed, the Wilcoxon signed rank test was used. The significance level was set at *p* < 0.05. All statistical analysis was carried out using R version 4.1.0 (The R Foundation for Statistical Computing, Vienna, Austria).

## 3. Results

A total of 89 hips (from 67 children) were reviewed, with 31 hips excluded due to inadequate radiographs. A total of 174 antero-posterior radiographs were included in the study. The statistical analysis yielded no significant difference in the age at surgery (*p* = 0.58), pre-operative GMFCS (*p* = 0.82) or gender (*p* = 0.57) between the included and excluded hips. The demographic data of the included patients are shown in Table 1.

### 3.1. Migration Percentage

**Figure 3:**
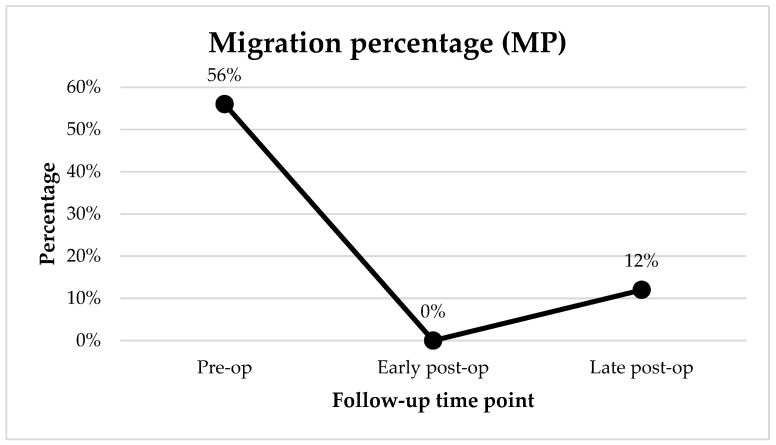
Median migration percentage at the pre-operative, early post-operative and late post-operative time points.

The migration percentage over time is shown in Figure 3. The median MP pre-operatively was 56% (IQR 46% to 85%). It improved significantly (*p* < 0.01) in the early post-operative follow-up to a median of 0% (IQR 0% to 15%). This reduction in hip displacement showed some progression in the late post-operative follow-up (*p* < 0.01) with a median MP of 12% (IQR 0% to 20%).

### 3.2. CE Angle

The median pre-operative CE angle was −3.25° (IQR −32.3° to 9.0°), indicating the poor coverage of the femoral head by the acetabulum. This improved significantly (*p* < 0.01) to a median of 28.8° (IQR 23.0° to 36.2°) at the early post-operative follow-up and remained stable with a median of 32.2° (IQR 27.7° to 40.8°) at the late post-operative follow-up.

### 3.3. Acetabular Index

The median pre-operative acetabular index was 30.4° (IQR 21.1° to 38.4°), indicating poor acetabular development. This decreased significantly (*p* < 0.01) to a median of 13.4° (IQR 3.4° to 18.2°) at the early post-operative follow-up and remained stable with a median of 12.1° (IQR 7.4° to 18.8°) at the late post-operative follow-up.

### 3.4. Sharp Angle

The mean Sharp angle pre-operatively was 49.9° (SD 7.1°). It improved significantly (*p* < 0.01) in the early post-operative follow-up to a mean of 37.2° (SD 8.2°). This correction was maintained in the late post-operative follow-up with a mean of 36.3° (SD 8.9°).

### 3.5. Pelvic Obliquity

There was no significant change in the pelvic obliquity across the follow-up time points.

### 3.6. Femoral Head Deformity

**Figure 4:**
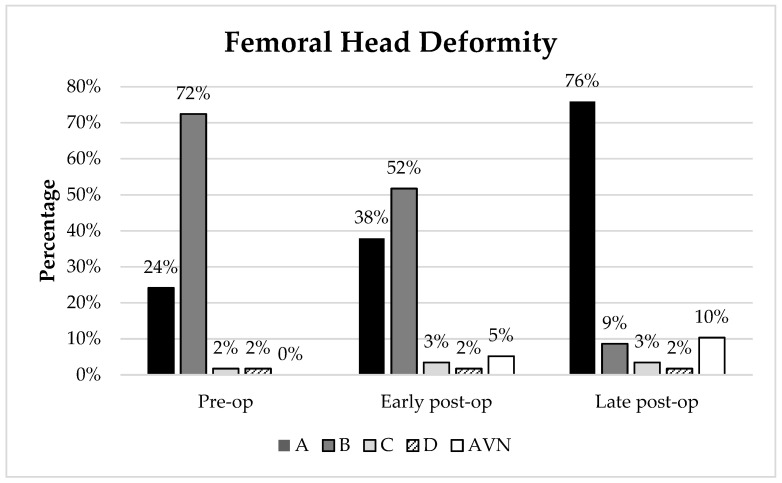
Femoral head deformity (in %) at the pre-operative, early post-operative and late post-operative follow-up. AVN = collapsed femoral head.

The number of hips according to femoral head deformity are shown in Figure 4 and Table 3. Pre-operatively, 14 hips (24%) were classified as grade A (rotund femoral head). This increased to 22 hips (38%) at the early follow-up and increased further to 44 hips (76%) at the late follow-up. The prevalence of a collapsed femoral head at the early post-operative follow-up was 5% (3 hips), and 10% (6 hips) at the late post-operative follow-up. There was no significant change in the median head sphericity between the pre-operative, early post-operative and late post-operative radiographs.

### 3.7. Neck Shaft Angle

The modified neck shaft angle reduced from a median of 170.3° (IQR 162.3° to 178.1°) to a median of 147.2° (IQR 143.1° to 156.0°) at the late post-operative follow-up.

### 3.8. Head Shaft Angle

The head shaft angle reduced from a median of 170.2° (IQR 161.5° to 176.8°) to a median of 134.1° (IQR 121.5° to 144.3°) at the late post-operative follow-up.

**Figure 5:**
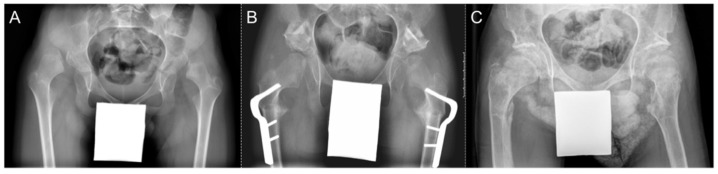
Radiographs of a patient with bilateral CP who underwent bilateral hip reconstruction surgery. (**A**) Pre-operative. (**B**) Early post-operative. (**C**) Late post-operative.

An example of a case of hip reconstruction surgery in a non-ambulatory (GMFCS V), 9-year-old male with bilateral CP is presented in Figure 5. Using the left side as an example, the MP was reduced from 100% to 0% after surgery. The reduction in the hip was maintained 5.5 years post-operatively with an MP of 18%. Prior to surgery, the femoral head had a grade B shape. This remodelled to a grade A shape at the early and late post-operative time points.

### 3.9. Orientation of Growth Plate

The median difference between the angle of the growth plate relative to the inter-teardrop line and the Sharp angle was −42.4° (IQR −51.1° to −35.1°) pre-operatively, indicating a poor congruency between the femoral head and the acetabulum. This improved post-operatively (*p* < 0.001) to a median of −0.5° (IQR −10.9° to 10.4°) in the early post-operative follow-up (*p* < 0.001), and was maintained in the late post-operative follow-up with a median of 1.7° (IQR −14.0 to 18.9°).

**Figure 6:**
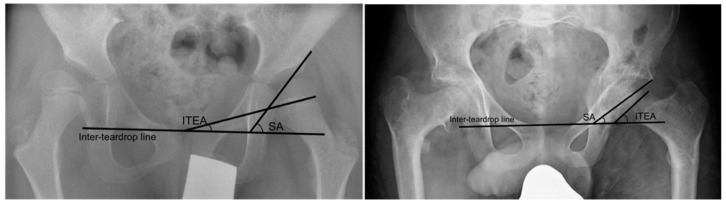
Pre-operative (**left**) and late post-operative (**right**) radiographs of a patient with bilateral CP who underwent bilateral hip reconstruction surgery.

An example of the change in growth plate orientation after hip reconstruction surgery in a non-ambulatory (GMFCS IV), 7-year-old male with bilateral CP is shown in Figure 6. In the pre-operative radiograph, the femoral head deformity was classed as grade B (flattening on one side). The difference between the ITEA and SA was 39.0°. In the late post-operative radiograph at 7.5 years follow-up, the femoral head remodelled to a grade A (spherical) head, with the growth plate orientation changing such that the difference between the ITEA and SA was markedly reduced to 4.6°.

## 4. Discussion

In this study, 58 hips from non-ambulatory children with CP that underwent hip reconstruction, with a median follow-up of 8.6 years, showed a significant improvement of most radiological measures. These improvements remained stable in the long term. In particular, the improvement in MP suggests that hip reconstruction is effective in reducing the hip and maintaining its placement in the long term. This supports previous findings that similarly support the long-term effectiveness of hip reconstruction surgery in maintaining the MP [14,16,29]. The gradual remodelling of the femoral head was shown in our cohort with the number of grade A femoral heads increasing over time.

The decreases in the acetabular index and Sharp angle found in our study suggest that there is improved acetabular shape after surgery. The CE angle also increased after surgery, suggesting a better coverage of the acetabulum over the femoral head. This replicates the findings of previous studies [30,31]. Although this is partly attributed to the pelvic osteotomy, some previous reports have suggested that femoral osteotomy itself may have some effects on acetabular development [32,33]. The modification to the femoral head may alter the distribution of forces within the acetabulum, resulting in the remodelling of the acetabulum.

When the hip joint is displaced, the femoral head may become flattened laterally, designated as a type B femoral head shape using the visual classification system. This may be due to forces acting on the femoral head from the lateral portion of the acetabulum and the surrounding muscles. Braatz et al. [29,30] reported that even when the femoral head is deformed pre-operatively, the plasticity of the hip joint allows for the remodelling of the femoral head from aspherical to spherical. Braatz et al. [29,30] also reported an improvement in congruency of the hip joint after surgery, with a further improvement at the long-term follow-up. The congruency was measured according to the difference between the medial and lateral joint gap. The hip joint was considered as spherical if the femoral head and the acetabulum fitted to circles. Min et al. [15] reported an improvement in the femoral head sphericity after hip reconstruction using the ratio between Mose circles fitted to the femoral head. However, Schlemmer et al. [16] reported no significant change in femoral head sphericity post-operatively using a similar measuring technique, which used the difference between the radii of fitted circles rather than a ratio. These studies did not differentiate between ambulatory and non-ambulatory children, who experience dissimilar forces acting on the hip joint due to differences in the weight-bearing status of the joint. In our study, we focussed on non-ambulatory children only, and found no significant change in femoral head sphericity between the pre-operative, early follow-up and late follow-up time points, when using the measurement of Mose hip ratios. We recognise that this measure of sphericity using only a two-dimensional anteroposterior view is limited as it does not take into account rotation or sphericity in other planes. This method of measuring sphericity is particularly troublesome when attempting to measure more severely deformed femoral heads, such as types C and D, where the ratio of circles may appear to rate the head as more spherical due to the shape of the deformity when viewed two-dimensionally. Ideally, a three-dimensional image of the femoral head would be required to determine the true sphericity. However, this is not always clinically practical.

We also interpreted the femoral head shape using a previously described classification system based on the visual inspection of the femoral head on anteroposterior radiographs [14]. Pre-operatively, 14 hips had femoral heads classified as type A, which indicates a more spherical femoral head. In the early post-operative follow-up point, this increased to 22 femoral heads. The number of femoral heads classified as type A increased further at the late post-operative follow-up to 44. This could suggest that there may in fact be some change in the femoral head shape that is not detected using the modified Mose technique. However, the use of this classification system also comes with limitations as it is based on qualitative visual inspection and its reliability and validity has not been tested. This indicates that even when the femoral head is deformed pre-operatively, hip reconstruction is still effective in reducing the hip, and resection of the femoral head may not be necessary as the reduction of the femur promotes reshaping towards a more spherical and congruent hip.

In a pilot study, Ali-Morell et al. [28] found some correlation between the increased MP and increased difference in the angle between the acetabulum and femoral growth plate. This finding suggests that the congruency between the femoral head and the acetabulum plays a role in the stability of the hip joint. In this study, we used two surrogate measures of hip joint congruency, the difference between the HEA and the AI and the difference between the ITEA and the SA. The latter measure was found to change closer to zero post-operatively and remained close to zero in the late post-operative follow-up, implying that the femoral growth plate becomes more parallel with the angulation of the acetabulum. This suggests that hip joint congruency improves after surgery and remains improved in the long term, perhaps contributing to maintaining the reduced hip joint. Correlating this with the improvement in MP post-operatively, and its stability in the long term follow-up, our findings corroborate those of Ali-Morell et al. [28] in proposing the importance of the acetabulum and femoral head congruency in maintaining the stability of the hip joint. These measures provide an additional way of considering joint congruency without being affected by difficulties in determining the sphericity of the femoral head. However, they are again limited by the inability to account for rotation or anteversion of the femur.

This study is limited by its retrospective nature and the lack of clinical outcome measurements, such as pain and quality of life. While changes in the radiographic features may not necessarily correlate with clinical improvement, increases in health-related quality of life, as measured using the Caregiver Priorities and Child Health Index of Life with Disabilities (CPCHILD), has been correlated with a lower MP on radiographs [34]. Hip reconstruction has also been shown to result in decreases in pain [14,31]. Additionally, only two-dimensional radiographs in one view were used, meaning the measurements could be affected by the anteroposterior dislocation of the hip, as well as rotation of the femurs or pelvis. However, three-dimensional CTs were not standard protocol during the study time period in order to minimise radiation exposure. In the patients nearing skeletal maturity, it was difficult to assess the growth plate orientation and position of the Hilgenreiner line due to the closure of the triradiate cartilage. There is also currently no validated classification of AVN or femoral head deformity in CP hips. Finally, our sample size was small and no control group was available to compare our results to children with other hip conditions.

## 5. Conclusions

In our cohort of patients, the number of grade A femoral heads increased from 24% pre-operatively, to 38% at the early post-operative follow-up, then 76% at the late post-operative follow-up. The existing mild femoral head deformities, grade A and B, are stable post-operatively and may even improve due to severe osteoporosis. Although non-ambulatory children do not have the same weight-bearing forces that promote bone remodelling in ambulatory children, it appears that they are still able to have a remodelling of the acetabulum and proximal femur after surgery. The relocation of the hip joint after surgery could promote remodelling through a better distribution of the forces acting between the acetabulum and femoral head. This remodelling continues in a long-term time frame. Additionally, the hips were successfully reduced after surgery, with MP reducing from a median of 56% pre-operatively to 0% at the early post-operative follow-up. The hips remained stable after a median time of 8.6 years with a median MP of 12%.

## Figures and Tables

**Table 1:** Patient demographics.

Number of hips	58
Number of children	45
Sex	23 female, 35 male
Side	32 left, 26 right
Topographic distribution	58 quadriplegia
Mean age at surgery (sd)	10.8 (3.1)
Median early post-operative follow-up in days (IQR)	120 (84 to 193)
Median late post-operative follow-up in years (IQR)	8.6 (6.4 to 13.6)
Pre-operative GMFCS	V = 34, IV = 24, III = 0, II = 0, I = 0

Radiological measurements are shown in Table 2.

**Table 2:** Radiological measurements.

Measure	Pre-Operative	Early Post-Operative	Late Post-Operative
Hip displacement			
MP	56% (46% to 85%)	0% (0% to 15%) *	12% (0% to 20%)
CE angle	−3.3° (−32.3° to 9.0°)	28.8° (23.0° to 36.2°) *	32.3° (27.7° to 40.8°)
Pelvic and acetabulum			
AI	30.4° (21.1° to 38.4°)	13.4° (3.4° to 18.2°) *	12.1° (7.4° to 18.8°)
Sharp angle	49.9° (SD 7.1°)	37.2° (SD 8.2°) *	36.3° (SD 8.9°)
PO	1.8° (−2.8° to 1.8°)	0.7° (−3.4° to 5.4°)	1.4° (−2.6° to 3.4°)
Sphericity and femoral head deformity			
Sphericity	0.85 (0.78 to 0.90)	0.83 (0.77 to 0.88)	0.84 (0.77 to 0.89)
mNSA	170.3° (162.3° to 178.1°)	-	147.2° (143.1° to 156.0°) *
HSA	170.2° (161.5° to 176.8°)	-	134.1° (121.5° to 144.3°)
HEA—AI	−23.9° (SD 17.3°)	26.9° (SD 18.9°) *	26.7° (SD 25.5°)
ITEA—SA	−42.4° (−51.1° to −35.1°)	−0.5° (−10.9° to 10.4°) *	1.7° (−14.0° to 18.9°)

Figures are provided as the mean or median with the interquartile range or standard deviation (respectively) in brackets. * significantly different to pre-operative.

**Table 3:** Femoral head deformity at the pre-operative, early post-operative and late post-operative follow-up, stratified by pre-operative deformity.

Pre-Operative FHD	Number of Patients	Post-Operative FHD	Early Post-Operative	Late Post-Operative
A	14	A	9	12
		B	5	1
		C	0	1
		D	0	0
		AVN	0	0
B	42	A	13	32
		B	25	3
		C	1	1
		D	0	0
		AVN	0	0
C	1	A	0	0
		B	0	1
		C	0	0
		D	1	0
		AVN	0	0
D	1	A	0	0
		B	0	0
		C	1	0
		D	0	1
		AVN	0	0

## Data Availability

The data presented in this study are available on request from the corresponding author. The data are not publicly available due to privacy of patients.
